# Experiences in elderly people with chronic obstructive pulmonary disease in relation to the use of long-term home oxygen therapy: a qualitative study about feelings attributed to therapy

**DOI:** 10.1186/s12890-022-01891-6

**Published:** 2022-03-19

**Authors:** Giovanna Hass Bueno, Claudinei José Gomes Campos, Egberto Ribeiro Turato, Ilma Aparecida Paschoal, Lucas Serra Valladão, Leticia Baltieri, Luiz Cláudio Martins

**Affiliations:** State University of Campinas (UNICAMP), Tessália Vieira de Camargo, 126, Cidade Universitária, Campinas, 13083-887 Brazil

**Keywords:** Qualitative research, Oxygen inhalation therapy, Aged, Pulmonary disease, Chronic obstructive, Body image, Family, Sadness

## Abstract

**Background:**

Elderly people are more likely to suffer severe chronic obstructive pulmonary disease (COPD) and require long-term home oxygen therapy (LTOT) as part of their treatment. LTOT has advantages such as improvement in symptoms, but there are also disadvantages such as physical barriers, psychosocial barriers and emotional challenges. The aim of this study is to understand the experiences of elderly people with COPD using LTOT with respect to their feelings attributed to therapy.

**Methods:**

Qualitative study. Seven semidirected interviews were conducted with patients with a confirmed COPD diagnosis who used LTOT and were treated at an outpatient service. The content analysis technique was applied with the support of WebQDA software 2.0.

**Results:**

Three categories emerged that were associated with the use of LTOT: (1) poor self-image; (2) feelings of sadness and (3) the impact of LTOT on others such as family and friends.

**Conclusions:**

LTOT in elderly people with COPD was associated with a poor self-image, feelings of sadness and impacted on others apart from the patient. When LTOT is prescribed, healthcare practitioners should proactively address these concerns to minimise the negative biopsychosocial experiences caused by LTOT.

## Background

The increase in life expectancy has led to an increase in the prevalence of noncommunicable chronic diseases [1]. According to the guidelines of the Global Initiative for Obstructive Lung Disease (GOLD), chronic obstructive pulmonary disease (COPD) is highly prevalent and may evolve with severe limitations on the lives of patients [2].

Patients in advanced stages of COPD often use long-term home oxygen therapy (LTOT) [2] in most cases for the rest of their lives [3]. Clinical studies have proven the benefits of LTOT, such as increased survival, improved exercise tolerance, reduced hospitalisations and improved mental status [3–7]. However, users recognise that there are also disadvantages, including physical and psychosocial barriers, as they become attached to the device and isolate themselves, and emotional challenges, such as fear and anguish about living in need of the machine. Furthermore, the treatment can be challenging [3, 8].

Elderly people face difficulties adapting to the use of LTOT in their lives and, in response, manifest changes in behaviour and feelings. It is necessary that health teams be prepared to address these life transitions, respecting the individuality of each patient. It is necessary not only to treat the chronic disease but also to take a holistic view of the patient.

The benefits of LTOT are already known, but it is necessary to understand how elderly people live with this therapy. Few research studies have focused on this topic. The aim of this study is to understand the experiences of elderly people diagnosed with COPD using LTOT with respect to their feelings attributed to the use of LTOT.

## Methods

A qualitative study was undertaken at the Pulmonology Clinic of UNICAMP Clinic Hospital (HC/UNICAMP), in a tertiary public university hospital located in the southeast region of Brazil.

### Population and sample

Inclusion criteria were patients seen at the outpatient service with a diagnosis of COPD according to the GOLD [2]; using LTOT for more than one year; older than 60 years; and communicating verbally and oriented with respect to time and space.

Sample closure occurred at the time of theoretical saturation; that is, the inclusion of new research participants was suspended when the data obtained were repetitive, redundant and failing to contribute significantly to the study [9]. The study included 7 patients who will be named sequentially from P1 to P7 in the body of the research.

### Data collection techniques and instruments

The data collection took place from June 2019 to August 2020. An introductory step for data collection was the setting in which the researcher observed and interacted with the local health team and patients. The collection instrument was improved through semidirected interviews.

Sociodemographic and historical data on the use of LTOT were collected during the interviews.

### Data analysis

Lawrence Bardin´s content analysis, which is “a set of communication analysis techniques, which uses systematic procedures and objectives to describe the content of messages”, was adopted as a methodological technique [10]. Content analysis is divided into three stages: preanalysis, material exploration and treatment of the results obtained and interpretation [10]. Data validation, performed by peers and judges, was carried out by two physicians, one a qualitative researcher and the other a pulmonologist, and two qualitative researcher nurses.

WebQDA Software version 2.0 was used as a support tool for the analysis of qualitative data with the objective of answering the questions that emerged from the investigation [11].

Two theoretical frameworks were used: Medical Psychology [12] and Psychosomatic Medicine [13, 14]. The COREQ criteria were used to report the method and results [15].

### Ethical issues

The project was approved by the Research Ethics Committee of the Faculty of Medical Sciences/UNICAMP under the number CAAE 87326218.7.0000.5404, approval number 2658702. Before participating in the research, the participants were informed that the interviews would be recorded and later analysed; upon agreement, they signed the Informed Consent Form.

## Results

Table 1 shows the biosociodemographic data. The duration of use of LTOT was 1–13 years, and the daily hours of use 18–24 h. Regarding the number of admissions and emergency room care visits in the 12 months prior to the interview, three had been hospitalised, and five had gone to the emergency room due to pulmonary problems.

**Table 1:** Biosociodemographic characteristics of the studied sample, Campinas 2020

Sociodemographic data						
Patient	Sex	Age (years)	Education	Job Position*	Religion	Marital status
P1	F	60	Illiterate	Housewife	Catholic	Lives with partner
P2	F	63	High School	Bath and grooming pet shop	Catholic	Lives with partner
P3	M	73	Basic Education	Works with cellulose	Atheist	Lives with partner
P4	M	63	Basic Education	Construction	Catholic	Lives with partner
P5	M	76	Basic Education	Truck driver	Catholic	Lives with partner
P6	F	60	High School	Administration at City Hall	Catholic	livEs with partner
P7	M	73	High School	Mechanic	Catholic	Lives with partner

*At the time of the interview, the participants were not working due to medical treatment

### Qualitative analyses

**Fig. 1:**
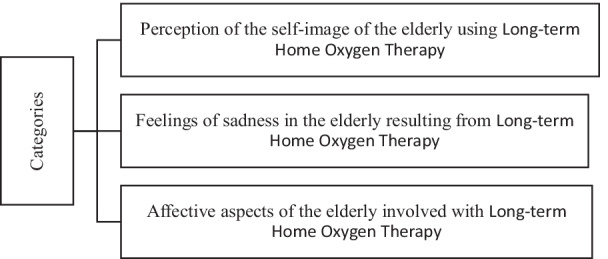
Study categories

Three categories emerged from the interviews (Fig. 1).

These analyses revealed 3 main categories, as shown in Fig. 1. The first category revealed was the perceived self-image of the elderly using LTOT. From the responses, it was evident that the use of LTOT can change the way the elderly perceive their self-image. Some patients felt dissatisfied with their physical appearance due to behavioural and psychological changes, whereas others were resigned to the treatment.

Perception of self-image is defined as the figuration of the body in the mind. P2 understood the need for therapy but reported this had not always been the case.

In the beginning, I used to walk around with this tube on the street, people used to make fun of me because of the equipment. I was being laughed at, but I lifted my head, continued, because it's for my own good. Before, I thought I was prettier. Today the oxygen catheter doesn't bother me anymore, but at first I felt ugly. My vanity changed, my hair wasn't short. I cut my hair very short like that, because, every time I am admitted to the ICU, my head gets sweaty. And just like that, I turned around, managed to wash my head in the sink and felt happier. Today, everyone knows me as the oxygen woman. In my city, I'm the only one. (P2).

For P2, short hair represented a little vanity. However, over time, she managed to adapt. In other words, the patient changed her way of thinking and changed her body model.

It was clear from the responses that the equipment associated with LTOT was often blamed for the reduced self-image. P6 reported feeling fat and P2 felt ugly, with both attributing this to the LTOT.

P6 felt independent; today, she has feelings of abnormality, highlighting how much patients consider themselves different and how much sadness this causes. She states that the therapy only brought a guarantee of life. It reinforces the idea that nothing is good, denying benefits and emphasising discomfort. There are ambivalent feelings.

I weighed 50 kg, today I'm 80 kg, I'm very fat. I cannot do anything. My self-esteem is down there. Sometimes, depending on the place, I don't go because I don't like people looking at me. I don't want people to look at me with pity. I used to have everything in place, I didn't know what a spare tire was. I didn't know what fat was. Today I am a bag of cellulite. I always had very high self-esteem, I guarantee it! Oxygen gave me the guarantee of being alive. That's all, because it didn't bring me anything else. Because I lived and worked, I was a normal person (P6).

In contrast to P2 and P6, P1, P4 and P5 accepted the LTOT. Whilst they valued beauty they seemed to accept their situation. Their personalities appeared to be less rigid, dealing less conflictingly with their own ageing and LTOT.

I like putting on an earring, putting on a string, dyeing my hair. After oxygen, that hasn't changed. (P1)

I'm not ashamed of using oxygen. (P4)

I already wear glasses straight away, the hose is just another thing on my face. (P5).

The second category identified in the qualitative analyses related to the feelings of sadness associated with the use of LTOT. Participants reported social isolation, interruptions at work, difficulty accepting the LTOT and suicidal ideation. There were also negative feelings, lack of interest and desire, sadness and frustration in life plans.

In the cases of P4 and P6, not doing activities is experienced as a loss of self-identity. Their work characterised who they were and the place they occupied in the community in which they live. P4 suffers from diagnosis and treatment, reaching the point of wanting to take his own life.

I had to stop working, because I worked with construction, and I cut tile and stone, leaving a lot of dust, preparing the cement, so the doctor told me to stop. I went to make soap, and the doctor ordered me to stop because of the smell. Then I went to make handicrafts, and depending on the handicraft, I had to stop, when it comes to wood, sanding, the dust and smells. Painting these things, stopped everything. It´s complicated! I suffered too much, it's crazy. I wanted to commit suicide. But today, it's going. They always told me there was no cure. I've tried to take poison, but I didn't get to take it, when I put the bottle close to my mouth, it released a white smoke and I fell. Then the neighbour ran there and helped me, it was crazy. There were days when I wanted to end everything, end life, really (P4).

A life of 100%, half of it, I vegetate. I don't feel like going out, doing anything. I earned very well, I worked, travelled, and today I can't do that anymore because I can't stand it. (P6).

The suffering of P2 is clear. She created an image in her mind that oxygen prevents her from going to the beach or even living in a coastal city. The life plan of growing old on the beach is interrupted, leaving her hopeless.

I have always wanted to see my son married, and go to work on the beach, live on the beach, but because of oxygen, I didn't go. That was always my dream. How am I going to walk with oxygen in the sand? (P2).

Feelings of melancholy and despondency were found in patients P1 and P5.

We don't feel like doing anything. It's all over, you don't feel like eating. I got depressed. I went into a very strong depression. (P1)

We get more depressed, right? I get up and lie down alone. I got more depressed after the oxygen. Discouragement can sometimes kill the person. (P5)

The third category revealed in the qualitative analyses related to the effect of LTOT on people apart from the actual patient. Patients reported feeling safe when having support from family members and revealed their concerns, anxiety, changes in habits in the life of the user and its impact on their family members.

P1’s husband comments that he will take care of her until the end of her life, showing contentment and affection.

My husband said 'I will take care of you until the last minute and you take care of me'. Will you give up? No. That's why I'm happy, I'm happy. (P1)

Some participants reported that their partner stopped doing something to help them. For example, the partner of P4 stopped smoking to enable her to improve her husband's health.

It's been 20 years since I stopped smoking, but I still feel a lot of desire when I smell it. My wife smoked, she quit because of me. (P4)

The attitudes of the family members of P6 and P7, of union and generousity, demonstrated that the patient is not alone and helped the patient feel comfortable to face all imposed changes naturally.

Now I am the daughter and they are the mother. All the time calling, all the time picking me up. It even bothers me a little, use gel alcohol, doesn't go there. Too much worry, wow!!! My whole family has changed a lot, we all got very close. (P6).

My children were very worried when I came home with oxygen, my wife too. They made a scheme at home, assembled everything, removed things from the place, put the cylinders, assembled everything to welcome me back. They thought it was a seven-headed beast, then they realised it wasn't. My friends were also worried, asking about me. Until one day, I went on the sidewalk, sat together with the oxyge, so everyone could see what I was using and how it was. Then everyone saw it and thought it was normal. (P7).

Family members became more concerned and zealous, as in cases P3 and P5.

My children are more on top of me, telling me to use oxygen. (P3)

My wife was more worried. She wants to go out somewhere, I say "go", she says "if you don't go I won't (P5)

**Fig. 2:**
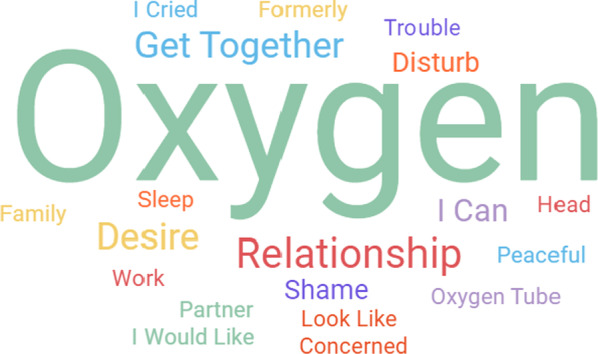
Word Cloud showing the most frequent terms in the participants' interviews

The Word Cloud, a tool used to highlight the main words from the interviews, is showing the most frequent terms in the participants' interviews in Fig. 2 which clearly indicates that oxygen is at the heart of these patients' lives. Words such as shame, image, and oxygen catheter show that LTOT causes changes in body representation. A term that indicates sadness, such as crying, is related to a feeling resulting from the therapeutic process. Family, relationship, concern, and partner are related to affective changes after the treatment is prescribed.

## Discussion

The aim of this qualitative study was to investigate the experiences of elderly people diagnosed with COPD using LTOT with respect to their feelings attributed to the use of LTOT. Three categories were revealed: the negative impact of LTOT on self-image, feelings of sadness associated with the use of LTOT and the effect of LTOT on family and carers. The words often used by participants during the interviews, highlights the visibility of LTOT, making the patient feel different or an invalid, leading to embarrassment [16].

Comparing our results to previous research, our findings that LTOT negatively impacted on self-image is consistent with authors as Kristina et al. [17], Janice and Colleen [18] and Hanneke et al. [19]. The breathlessness felt by the patient will lead to a loss of control of his own body, affecting his self-image [17]. The unpredictable situations caused by sudden desaturation will lead to a daily struggle between emotional reactions and the perception of the breathing rhythm changes, exposing his body to the social environment [17, 18]. In other words, the psychological condition is responsible for this lack of control, causing the patient to feel ashamed to attract even more attention beyond their apparent condition, and as a result, their appearance and self-image is unsettled and put aside. Self-care may be compromised, leading to embarrassment, worsening self-image, decreasing pleasure and worsening isolation for these patients [19].

The feelings of sadness associated with the use of LTOT that were identified in our study are comparable to those reported previously by Kristina & Britt-Marie [20], Margaret [21] and Hanneke et al. [19]. Patients see only their weaknesses [20], they feel trapped in their lungs [19], as symptoms vary at any time, resulting in restrictions in daily life and social isolation [20]. Patients manifest negative feelings such as frustration, hopelessness and low self-esteem, which may be associated with anxiety and depression [21]. In some cases, the sadness is so intense that individuals consider committing suicide [21].

The impact of the need for LTOT on people apart from the patient, such as family, friends, carers has been identified in previous research such as Kristina & Britt-Marie [20], Margaret [21] and Janice & M.Colleen [18]. Families are deeply affected by changes in the patient's life [18, 20]. The roles of family members change, which can affect communication and harmony, causing conflicts, stress and worsening tension in relationships [21].

The finding of this study reinforces the need to educate patients receiving LTOT regarding the potential benefits such as improvement of dyspnoea and clinical conditions but also the challenges of LTOT, such as the psychosocial barriers [8].

Coping with the difficulties imposed by LTOT and reaching acceptance is not easy; to do so, a patient goes through several stages, such as denial and isolation, anger, bargaining and depression [22]. In this process, suicidal behaviour can arise. Melancholy is intense, and device dependence and sadness are pathological. The patient needs to feel cared for, protected, and valued by the health team, not rejected or abandoned. Touching the subject of suicidal behaviour can be a way of the patient taking action, for feeling misunderstood and not being taken seriously [23]. As is clear from our results, the family and friends of LTOT users also experience changes as a result of the LTOT [8, 24].

The issues raised in our study, such as a reversal of the roles of parents and children, and monitoring of the patient shows that both patients and family members need to be very clear that the disease implies readaptation of social and family roles [6]. The importance of approaching the family is highlighted as an integral part of the patient's treatment, offering comprehensive patient care. Healthcare professionals need to create skills to understand family dynamics and establish a trusting relationship to increase the likelihood of adherence to LTOT [25].

Our study had a number of limitations. In particular, we believe that the environment of the interviews was adversely affected by the COVID-19 pandemic as it impacted on the relationship between interviewer and patient.

## Conclusions

This study found that LTOT in the elderly had a negative impact on self-image and led to feelings of sadness and discouragement. The use of LTOT also impacted more widely than just the patient, with family, carers and friends also affected. An awareness of these issues when prescribing LTOT may help minimise the negative aspects of the biopsychosocial experiences that the therapy causes in users and family members.

## Data Availability

The datasets used and/or analysed during the current study are available from the corresponding author.

## Abbreviations

COPD: Chronic Obstructive Pulmonary Disease
LTOT: Long-term Home Oxygen Therapy
GOLD: Global Initiative for Obstructive Lung Disease
HC/UNICAMP: UNICAMP Clinic Hospital
CAAE: Certificate of Presentation of Ethical Appreciation
O_2_: Oxygen
